# Whey protein and essential amino acids promote the reduction of adipose tissue and increased muscle protein synthesis during caloric restriction-induced weight loss in elderly, obese individuals

**DOI:** 10.1186/1475-2891-11-105

**Published:** 2012-12-11

**Authors:** Robert H Coker, Sharon Miller, Scott Schutzler, Nicolaas Deutz, Robert R Wolfe

**Affiliations:** 1 Center for Translational Research in Aging and Longevity, Little Rock, AR, USA; 2 Department of Geriatrics, University of Arkansas for Medical Sciences, and Healthspan, LLC, Little Rock, AR, USA

**Keywords:** Obesity, Fat, Protein

## Abstract

**Background:**

Excess adipose tissue and sarcopenia presents a multifaceted clinical challenge that promotes morbidity and mortality in the obese, elderly population. Unfortunately, the mortality risks of muscle loss may outweigh the potential benefits of weight loss in the elderly. We have previously demonstrated the effectiveness of whey protein and essential amino acids towards the preservation of lean tissue, even under the conditions of strict bedrest in the elderly.

**Methods:**

In the context of caloric restriction-based weight loss, we hypothesized that a similar formulation given as a meal replacement (EAAMR) would foster the retention of lean tissue through an increase in the skeletal muscle fractional synthesis rate (FSR). We also proposed that EAAMR would promote the preferential loss of adipose tissue through the increased energy cost of skeletal muscle FSR. We recruited and randomized 12 elderly individuals to an 8 week, caloric restriction diet utilizing equivalent caloric meal replacements (800 kcal/day): 1) EAAMR or a 2) competitive meal replacement (CMR) in conjunction with 400 kcal of solid food that totaled 1200 kcal/day designed to induce 7% weight loss. Combined with weekly measurements of total body weight and body composition, we also measured the acute change in the skeletal muscle FSR to EAAMR and CMR.

**Results:**

By design, both groups lost ~7% of total body weight. While EAAMR did not promote a significant preservation of lean tissue, the reduction in adipose tissue was greater in EAAMR compared to CMR. Interestingly, these results corresponded to an increase in the acute skeletal muscle protein FSR.

**Conclusion:**

The provision of EAAMR during caloric restriction-induced weight loss promotes the preferential reduction of adipose tissue and the modest loss of lean tissue in the elderly population.

## Introduction

The elderly population is particularly prone to the accelerated loss of muscle mass or sarcopenia with advancing age. Sarcopenia is complicated by the combined influence of physical inactivity and poor nutrition that often accompanies the aging process [1]. The condition of sarcopenia is inextricably linked to loss of functional capacity, and increased risk of morbidity and mortality [2-5]. While the precise etiology of sarcopenia has yet to elucidated, recent evidence points to a overall reduction in the anabolic response of skeletal muscle to the consumption of mixed macronutrient intake [6,7], potentially contributing to chronic, progressive reduction of muscle mass with advancing age.

Combined with the challenge of sarcopenia, the prevalence of obesity among elderly adults has increased dramatically [8]. The concomitant development of sarcopenic obesity results in the formation of overlapping health problems that combine the negative influences of inflammation, frailty and metabolic syndrome [9,10]. Moreover, caloric restriction-induced weight loss that is often utilized to dampen the negative consequences of metabolic syndrome may be considered counterproductive when measured against weight loss-induced acceleration of sarcopenia [11].

While moderate increases in dietary protein intake beyond the Recommended Daily Allowance (RDA) have been suggested to enhance anabolic activity in skeletal muscle [12], amino acid consumption have also been shown to facilitate an increase in muscle protein synthesis [13,14]. In addition, supplementation of high quality protein twice a day promoted an increase in lean body mass, strength and functional capacity in elderly volunteers with concomitant changes in diet or activity [15]. The development of the most potent formulation of whey protein and amino acid supplementation would potentially protect against the negative consequences of sarcopenic obesity in the elderly.

To meet our aims, we chose a caloric restriction paradigm designed to elicit 7% weight loss and decrease metabolic risk [15]. Our overriding hypothesis was that the supplementation of our whey protein + essential amino acid meal replacement (EAAMR) formulation will be required to promote the optimal reduction of adipose tissue while preserving lean tissue in obese, elderly adults. We also proposed that the changes in body composition would occur in conjunction with an increase in the skeletal muscle protein fractional synthetic rate (FSR) to EAAMR compared to competitive meal replacement (CMR).

## Methods

### Recruitment and screening

We recruited and enrolled healthy males and females, ages = 65-80 years of age and of all races and ethnic backgrounds for a caloric restriction-based weight loss intervention designed to promote 7% weight loss. All volunteers were weight stable and were not participating in a weight loss or exercise program at the time of enrollment. Volunteers refrained from alcohol consumption (24 h) and intense physical activities (72 h) prior to pre- and post-weight loss study visits. Volunteers also reported in the fasted state beginning at 20:00 h the day prior to pre- and post-weight loss study visits.

In conjunction with the consumption of solid food in both groups, subjects were randomized to one of two groups that would consume either EAAMR or a CMR five times/day. Volunteers were recruited through the conspicuous placement of IRB-approved flyer throughout the community, and completed a telephone screening. Potential subjects were then asked to report for a visit to our clinic, where they were then consented.

We excluded any patient with a chronic inflammatory condition or malignancy. We also excluded any individual who could not discontinue the use of aspirin or other anti-coagulant medication. In addition, volunteers with a history of diabetes, cardiovascular disease or recent history of cancer (within 3 years) were also excluded. We also excluded any individual whose body weight had changed more than 10 pounds in the previous 3 months. Due to the nature of the weight loss intervention, we excluded all volunteer with allergies to milk products.

It would have been impractical to exclude all patients taking medications, and we could not ethically discontinue all medications for the duration of the intervention. As a result, we permitted the concomitant use of HMG CoA-reductase inhibitors, thiazide diuretics, beta-1 selective blockers, clonidine, antidepressant selective serotonin reuptake inhibitors, and estrogen replacement/oral contraceptives.

### Weight loss study design

All medical screenings included a baseline blood sample collection for a lipid and metabolic panel. Based on eligibility, volunteers were randomly assigned to one of two groups: 1) 7% weight loss with EAAMR, or 7% weight loss using CMR. Both groups consumed 5 servings of a meal replacement (170 kcal x 5 servings/day) and ~400 kcal/day of solid food that yielded ~1250 kcal/day. Regardless of group assignment, the total kcal/week remained constant throughout the intervention to ensure a high level of compliance and allow for consistent, reliable reductions in the amount of weight lost. Obese, elderly volunteers in both groups (EAAMR or CMR) completed one visits that included a stable isotope experimental session described below. Following the completion of the experimental testing sessions, all volunteers were instructed to consume a low calorie diet for 8 weeks. During this weight loss intervention, volunteers consumed ~1250 kcal/day consisting of solid food and liquid supplements. Although the time course of the caloric restriction phase may seem somewhat overbearing from a logistical standpoint, the difficulty entailed was dramatically reduced through the use of pre-packaged meal replacements either in the form of EAAMR or CMR that required no preparation. In addition, we suggested solid food products preferred by clients in the UAMS weight loss clinic that achieve optimal compliance and consistently provide predictable weight loss. Body weight and body composition was measured weekly by our clinical staff. Any volunteer that failed to lose weight on two subsequent visits was removed from the study for noncompliance to the protocol.

Experimental Paradigm. Prior to initiation of the weight loss intervention, volunteers in both groups completed a testing session designed to examine the influence of EAAMR and CMR on skeletal muscle protein FSR. Subjects were instructed to fast after 2200 hrs, with water permitted. On the day of the testing session, subjects reported to the study site, and two intravenous catheters were inserted by our registered nurse. One catheter was utilized for the infusion of stable isotopes, while the other was utilized for blood sampling. A primed (2 μmol/kg), constant (0.07 μmol/kg/min) infusion of ring- [13]C_6_-phenylalanine began after obtaining a background blood sample and the isotopic infusion was maintained for 6 hours. Blood was sampled at 20 min intervals to determine tracer enrichment and blood amino acid levels. A tissue biopsy of the vastus lateralis muscle was taken under local anesthesia at t = 60, 180 and 240 min [16]. Biopsies were utilized for the determination of skeletal muscle protein FSR by tracer methodology [17]. After the initial biopsy at t = 60 min, subjects were provided with a single dose of EAAMR or CMR. At t = 180 and t = 240 min, two biopsies were taken to evaluate the influence of EAAMR or CMR on the incorporation of the phenyalalnine tracer into the muscle.

We have previously demonstrated that characterization of this acute response to EAA reflects the 24 h response, and that skeletal muscle protein FSR remains constant for the entire 8 hours if no drink is given [18]. While both products were presented in a tetrapak, and in a palatability format that precluded specific determination of either product by the volunteer, the knowledge of the product alone would not have influenced skeletal muscle protein FSR. Following the completion of the infusion protocol and biopsy, catheters were withdrawn, the biopsy site was dressed, and after consult with the study physician, the subject was released.

### Body weight and composition

We measured body mass, BMI, and body composition pre- and post-weight loss, and during each weekly visit. Total body mass was to the nearest 0.1 kg using an electronic scale (Ohaus Corp, USA). A GAIA 359 PLUS (Kyungsang Buk-do, KOREA) unit measured fat mass, lean tissue mass, soft lean mass and percent body fat using bioelectrical impedance analysis via the tetra-polar electrode method.

### Analysis of samples

#### Blood

The isotopic enrichment of L-[ring- [13]C_6_phenylalanine in blood was determined on an HP Model 5973 GCMS (Hewlett-Packard Co., Palo Alto, CA) by electron impact ionization and selected ion monitoring [17].

#### Muscle

Tissue biopsies of the vastus lateralis were immediately rinsed with cold saline, blotted, and frozen in liquid nitrogen. These tissue samples were stored at -80°C until processed. The TBDMS derivative was prepared for the intracellular free water as previously described 56, and analyzed by gas chromatography/mass spectrometry (GCMS) (Model 5973) using electron impact ionization. The protein-bound enrichment of phenylalanine was analyzed as previously described by GCMS [17].

#### Calculation of skeletal muscle protein FSR

Skeletal muscle protein FSR was calculated from the determination of the rate of tracer incorporation into the protein and the enrichment of the intracellular pool as the precursor:

$$\mathrm{FSR}=\left[\left(E_{p2}-E_{p1}\right)/\left(E_{M}t\right)\right]\cdot 60\cdot 100$$

where E_p1_ and E_p2_ are the enrichments of the protein-bound L-[ring- [13]C_6_phenylalanine at t = 60, 180, and 240 min. E_M_ represented the average intracellular L-[ring- [13]C_6_phenylalanine enrichments over the time of incorporation; and t is the time in minutes. The factors 60 and 100 were required to express skeletal muscle protein FSR in percent per hour. Skeletal muscle protein FSR was calculated from the biopsies at 60 and 180 min to determine fasting protein synthetic rate, and also from t = 180 min and t = 240 min to determine the effect of drink administration. The drink was given immediately after the 180 min biopsy and thus incorporation of the tracer from 180-240 min reflects acute nutritional response. From the skeletal muscle protein FSR and lean body mass determination the total change in skeletal muscle protein synthesis was estimated [18].

### Statistical analysis

Variables were summarized using means, standard deviations, medians and ranges. The groups were compared with respect to changes in adipose and lean tissue, and skeletal muscle protein synthesis as measured by skeletal muscle protein FSR, using Student two-sample t-tests. An alpha-level of 5% was used to determine statistical significance.

## Results

### Subjects

We enrolled 12 volunteers, and one subject dropped out due to their inability to comply with the protocol. Therefore, 11 obese women and men lost 7% of total body weight, and completed all aspects of the study. There were no baseline differences in age, BMI or percent fat, or the distribution of gender equity between the EAAMR and CMR groups (*p* > 0.05) (Table 1). Of these individuals, one of the individuals in the EAAMR group had undergone a hip replacement in 2003, and one of the individuals in the CMR group had been successfully treated for cancer in 1999. In addition, four out of the 11 individuals were taking simvastatin or nexium, and two of the 11 individuals were taking estradiol.

**Table 1:** Age and Anthropometrics (pre- and post- intervention

	**EAAMR**		**CMR**	
	**Pre WL**	**Post WL**	**Pre WL**	**Post WL**
Age	70 ± 2		68 ± 2	
Body weight (kg)	92.3 ± 3.9	84.5 ± 3.3*	91.4 ± 2.4	84.6 ± 2.4
Body mass index (kg/m^2^)	31.3 ± 0.5	29.1 ± 0.7	31.3 ± 0.5	30.0 ± 0.4
Percent Fat	41.8 ± 1.1	36.3 ± 1.1*	38.9 ± 1.5	37.5 ± 0.2

Values are means ± SEM. There were no significant differences in these variables at basekine. *Denotes a significant change from pre-to post-intervention (P < 0.05).

Baseline Metabolic and Lipid Parameters. There were no significant differences in the fasting glucose values between EAAMR and CMR (*p* > 0.05) (Table 2). In addition, there were no differences in triglycerides, total cholesterol, high density lipoprotein or very low density lipoprotein (*p* > 0.05). While there was a trend towards reduced low density lipoprotein in EAAMR, it was not significant (*p* = 0.10).

**Table 2:** Plasma glucose and lipid variables

	**EAAMR**	**CMR**
Glucose (mg/dl)	97 ± 4	103 ± 7
Triglycerides (mg/dl)	154 ± 22	115 ± 13
Total cholesterol (mg/dl)	168 ± 7	191 ± 9
High density lipoprotein (mg/dl)	47 ± 4	47 ± 5
Very low density lipoprotein	31 ± 4	23 ± 3
Low density lipoprotein (mg/dl)	86 ± 9	121 ± 12

Values are means ± SEM. There were no significant differences in these baseline variables (P > 0.05).

### Anthropometrics

**Figure 1:**
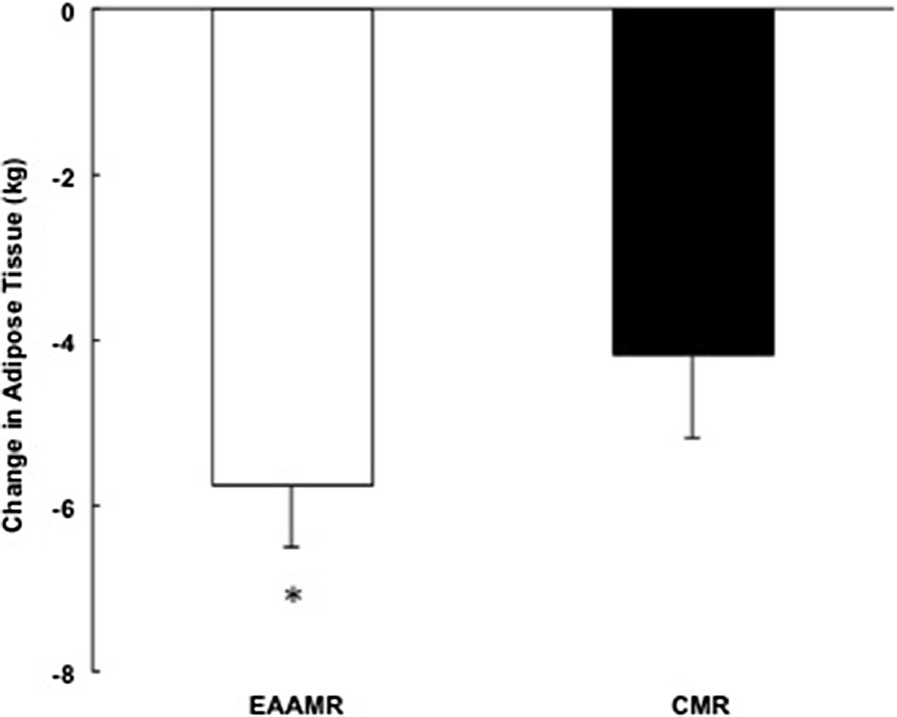
**Change in adipose tissue in EAAMR and CMR following weight loss.** * Denotes significance difference between groups.

**Figure 2:**
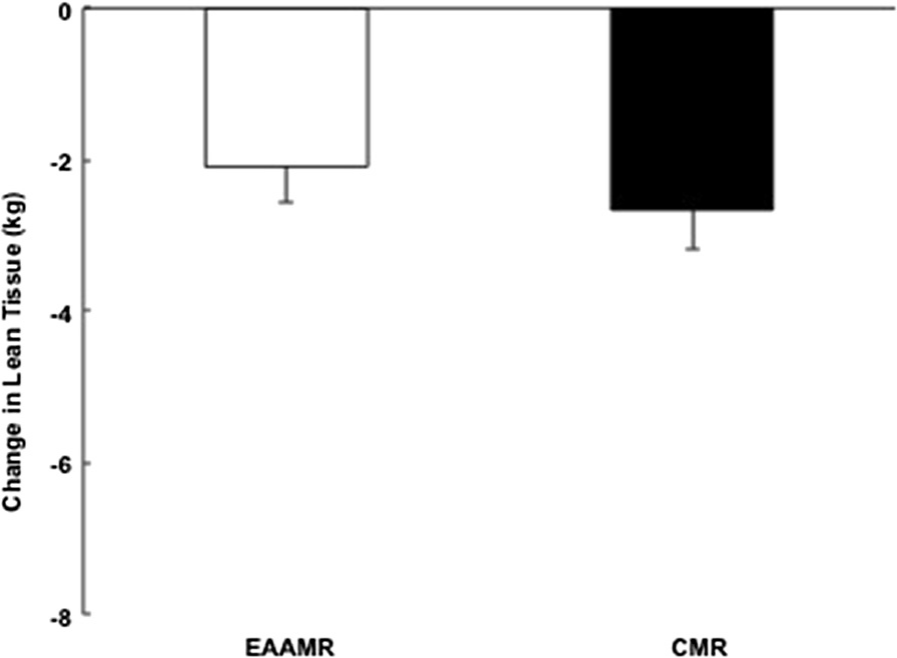
Change in lean tissue in EAAMR and CMR following weight loss.

Relative to the caloric restriction-induced weight loss interventions, there was a significant and similar 7% reduction in body weight in EAAMR and CMR (Table 1). While there was no difference in absolute amount of weight loss, EAAMR promoted a greater reduction in adipose tissue compared to CMR (*p* < 0.05) (Table 1) (Figure 1). The consumption of the EAAMR also seemed to foster greater preservation of lean tissue compared to CMR. However, the difference between EAAMR and CMR was not significant (*p* = 0.26) (Figure 2).

### Plasma and muscle phenyalanine enrichments

Basal plasma phenyalanine enrichments were not different between EAAMR and CMR (*p* > 0.05). Upon ingestion of each product, muscle phenyalanine enrichments increased (*p* < 0.05) dramatically in EAAMR compared to no change with CMR (*p* > 0.05).

**Figure 3:**
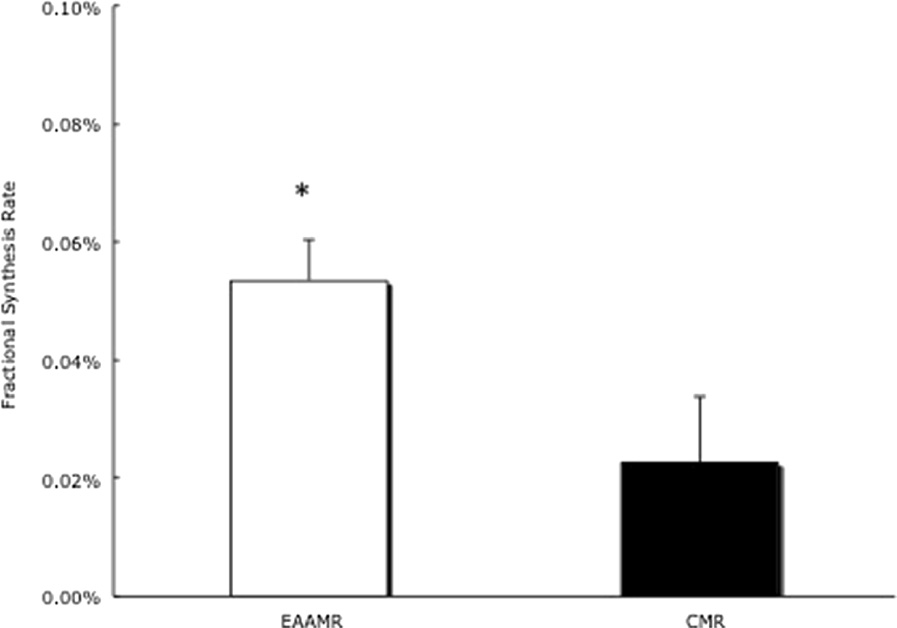
**Change in feeding induced skeletal muscle FSR in EAAMR and CMR following weight loss.** * Denotes significance difference between groups.

Skeletal Muscle Fractional Protein Synthesis. Skeletal muscle protein FSR increased by 0.0142 ± 0.0154% in CMR (Figure 3). In contrast, there was a greater increase (ie., 0.0534 ± 0.0069%) in skeletal muscle protein FSR in EAAMR (*p* < 0.05) (Figure 3).

## Discussion

Our experimental approach allowed us to determine whether consumption of EAAMR was more effective than CMR in promoting the concomitant reduction of adipose tissue and preservation of lean tissue during caloric restriction-induced weight loss in elderly, obese individuals. Due to the relative importance of in vivo energetics of muscle protein turnover towards energy expenditure [19], we also evaluated the acute influence of EAAMR compared to CMR on skeletal muscle protein FSR. Using a 1200 kcal/day, 8 week caloric restriction-weight loss paradigm, there was a 30% greater loss of adipose tissue with EAAMR compared to CMR. While the sample size may have affected the outcome, consumption of EAAMR seemed to favor the preservation of lean tissue but was not significant with the limited number of participants [20]. Nonetheless, skeletal muscle FSR was significantly higher with the acute consumption of EAAMR versus CMR, and may have been responsible for the preferential loss of adipose tissue with EAAMR.

The macronutrients contained in a single serving of EAAMR and CMR are described in Table 3. It is important to mention that the total caloric value for a single serving of EAAMR and CMR was ~170 kcal. The amount/type of carbohydrate was identical in both formulations. Also, the total amount of fat was 4 grams in EAAMR and 3 grams in CMR with the proportion of saturated fat being 1 gram in both EAAMR and CMR. The primary differences between EAAMR and CMR were the total amount/type of intact protein and the total amount and formulation of essential amino acids. While EAAMR contained 7 grams of intact protein from whey protein, CMR contained 14 grams intact protein derived from sodium casenate and calcium casenate. Leucine comprises approximately 40% of the EAAs, in accord with our research defining the optimal formulation of EAAs for elderly [13,16,21]. We have also previously demonstrated greater accrual of muscle protein with whey protein [22], and we proposed that the combination of whey protein plus 6 grams of our proprietary essential amino acid formulation would be the most effective approach against sarcopenic obesity. The total amount of essential amino acids derived from intact protein and the essential amino formulation was 9.7 grams in EAAMR. The total amount of essential amino acids derived from intact protein was 3.5 grams in CMR. The description highlights the fundamental differences between EAAMR and CMR and the rationale for the source of intact protein and importance of the essential amino acids formulation.

**Table 3:** Nutrition Facts for EAAMR and CMR

	**EAAMR**	**CMR**
Calories	170	170
Total Fat (grams)	4	3
Saturated Fat (grams)	1	1
Trans Fat (grams)	0	0
Cholesterol (mg)	5	5
Sodium (mg)	220	220
Potassium (mg)	460	460
Total Carbohydrate	22	22
Fiber (grams)	1	1
Sugars (grams)	17	17
Protein (grams)	7	14
Essential amino acid formulation	6	0

There are several factors that should be considered when comparing the effects of meal replacements on changes in body composition and the energetics of skeletal muscle in the context of weight loss. Typically, total energy expenditure is represented by the sum of resting energy expenditure, the thermic effect of food, and the energy related to activity [23]. Given that that the experimental protocol was identical in both groups except for the nutrient profile of the meal replacements, we can make the assumption that basal energy expenditure and activity related energy expenditure were not responsible for the differences between EAAMR and CMR. Other factors such as the administration of the meal replacements, timing sequence of the protocol, the characterization of the subjects, and the length of the protocol could have also influenced the results related to body composition. These factors were either well controlled by the experimental design or were not significantly different between groups. Therefore, the impact of the nutrient profiles (ie., type of protein and formulation of essential amino acids) was likely responsible for the greater loss of adipose tissue through differences in diet-induced energy expenditure or the impact of muscle loss sparing on overall energy expenditure.

While we were not able to achieve statistical significance in terms of EAAMR promoting enhanced preservation of lean tissue, the sparing influence of muscle loss might have been demonstrated with a larger sample size. This remains an important issue. Acute administration of EAAMR did promote a significant increase in skeletal muscle protein FSR compared to CMR. Assuming that the energy cost of protein synthesis is 3.6 kJ/g and the baseline GAIA-derived lean tissue mass was 56.4 kg for EAAMR and 54.4 kg for the CMR, we can extrapolate that the overall energy discrepancy between the two groups was roughly equivalent to 27,170 kcal or 3.5 kg of weight loss across the entire caloric restriction-based weight loss paradigm. Based on the amount of total lean mass in each group, this value takes into account a consistent intervention structure of five servings/day across an eight week period. In short, these calculations suggest that differences in the source of intact protein/formulation of EAA may have a significant influence on diet induced energy expenditure that coincides closely with the greater reduction of adipose tissue in EAAMR compared to CMR.

Despite the greater stimulation of skeletal muscle protein FSR in EAAMR, these individuals still lost 2.1 ± 0.5 kg of lean tissue. With an estimated protein intake of 90 grams/day, this might seem confusing since the recommended dietary allowance (RDA) of 0.88 kg^-1^· d^-1^ would equate to 50 grams of protein in the EAAMR group [24]. On the other hand, the RDA for protein has been shown to be inadequate for the maintenance of lean body mass in the elderly population [25]. Therefore, the optimal amount of protein intake in the elderly population remains uncertain [26], especially in the context of caloric restriction. The underlying reason for the lack of precise information when it comes to protein intake during weight loss in this population may be largely due to the fact that the protein RDA levels were determined by studies using nitrogen balance in younger individuals [27]. In order to attenuate the acceleration of sarcopenia, the amount/type of protein and/or formulation of essential amino acids must be elucidated to promote efficacious caloric restriction-based weight loss in the elderly.

For the effective treatment of sarcopenic obesity, it might be argued that exercise training should be utilized instead of or in conjunction with caloric restriction [28]. In fact, we have also recently demonstrated greater reduction of adipose tissue through the use of exercise-induced weight loss compared to caloric restriction [29]. There was also a greater preservation of lean tissue with exercise-induced weight loss compared to caloric restriction-induced weight loss [30]. Unfortunately, individuals at risk for metabolic disease may be limited in their ability to even meet the guidelines for physical activity much less induce weight loss through exercise training [30]. In fact, only 30% of young, healthy Americans currently utilize exercise as an efficacious strategy for weight loss [31]. These issues are compounded by the difficulties entailed with maintaining exercise compliance [32]. Therefore, caloric restriction-based weight loss provides a potential avenue for an improvement in metabolic health status and may influence functional status as well due to the inverse relationship between obesity and physical activity [33].

Weight loss in the obese, elderly population is typically contraindicated due to difficult, clinical conundrum of sarcopenic obesity. While the results of the present study do not suggest that EAAMR would be sufficient to completely prevent the reduction of lean tissue during caloric restriction-induced weight loss, we were able to demonstrate the preferential reduction of adipose tissue with 7% weight loss. Moreover, the acute anabolic response to the ingestion of EAAMR was directly linked to increased diet-induced caloric expenditure that may have responsible for the increased loss of adipose tissue. Due to results of the current investigation, we anticipate that improvements in insulin sensitivity derived from the preferential loss of adipose tissue will maximize the anabolic efficiency to nutrition. Based on earlier studies where we have examined the influence of EAA on muscle protein synthesis [21], a two-sample t-test would have 80% power to detect effect sizes as small as 0.566 in 35 volunteers. It is also important to mention that the individuals in the current study were still deficient in terms of the their total protein intake in the context of caloric restriction, and we are currently revising our formulation to address this issue. In conclusion, we propose that high quality protein + essential amino acids represent a critical variable in the preservation of lean tissue and augmentation of adipose tissue reduction in the elderly population.

## Abbreviations

EAAMR: Essential amino acid meal replacement; CMR: Competitive meal replacement; RDA: Recommended daily allowance; FSR: Fractional synthetic rate; GCMS: Gas chromatography/Mass spectrometry; BMI: Body mass index.

## Competing interests

The authors’ declare that they have no competing interests.

## Authors’ contributions

RHC conceived the study, and participated in its design and coordination, performed the statistical analysis and drafted the original manuscript. SM and SS participated in the design and coordination of the study. ND participated in the design and coordination of the study, and served as the study physician. RW provided key insight into the conception of the study and edited the final draft of the manuscript. All authors read and approved the final manuscript.
